# The Walking-Induced Transient Hack Concept Is Valid & Relies on a Transient Early-Exercise Hypoxemia

**DOI:** 10.1371/journal.pone.0062651

**Published:** 2013-05-03

**Authors:** Antoine Bruneau, Mathieu Feuilloy, Corinne Dussaussoy, Frédéric Gagnadoux, Georges Leftheriotis, Pierre Abraham

**Affiliations:** 1 L’Université Nantes Angers Le Mans, Centre Hospitalier Universitaire d’Angers, Laboratory for Vascular Investigations, Angers, France; 2 Graduate School of Engineering, Angers - Laboratoire d’Acoustique de l’Université du Maine – Unité Mixte de Recherche, Centre National de la Recherche Scientifique U6613, Angers, France; 3 Centre Hospitalier Universitaire d’Angers, Department of Respiratory Diseases, Angers, France; 4 L’Université Nantes Angers Le Mans, Institut National de la Santé et de la Recherche Médicale U1063, Angers, France; 5 L’Université Nantes Angers Le Mans, Unité Mixte de Recherche, Centre National de la Recherche Scientifique U6214, and Institut National de la Santé et de la Recherche Médicale, U1083, Angers, France; University Hospital Vall d’Hebron, Spain

## Abstract

**Background:**

Decreased arterial oxygen pressure obtained at peak exercise is strong evidence of walking-induced hypoxemia, assuming that the lower pressure occurs just before exercise is stopped. Using empirical predefined models and transcutaneous oximetry, we have shown that some patients reporting exercise intolerance show a minimal value at the onset of walking and a post-exercise overshoot. These changes are referred to as transcutaneous “walking-induced transient hacks”.

**Methods:**

In 245 patients, walking-induced transcutaneous oxygen pressure changes in the chest were analyzed using observer-independent clustering techniques. Clustering classes were compared to the profile types previously proposed with the cross-correlation technique. The classifications of patients according to both approaches were compared using kappa statistics. In 10 patients showing a hack on transcutaneous oximetry, we analyzed the results of direct iterative arterial sampling recorded during a new walking treadmill test.

**Results:**

Clustering analysis resulted in 4 classes that closely fit the 4 most frequently proposed empirical models (cross-correlation coefficients: 0.93 to 0.97). The kappa between the two classifications was 0.865. In 10 patients showing transcutaneous hacks, the minimal direct arterial oxygen pressure value occurred at exercise onset, and these patients exhibited a recovery overshoot reaching a maximum at two minutes of recovery, confirming the walking-induced transient hypoxemia.

**Conclusions:**

In patients reporting exercise intolerance, transcutaneous oximetry could help to detect walking-induced transient hypoxemia, while peak-exercise arterial oximetry might be normal.

## Introduction

As a result of increased ventilation and ventilation-to-perfusion ratio improvement during exercise, the expected normal response to walking of arterial oxygen pressure (pO_2_) is an increase from the resting value and a decrease in the recovery period. Assuming that transcutaneous pO_2_ changes at the chest level mimic arterial pO_2_ changes [1], [2], transcutaneous pO_2_ is expected to increase at walking onset and decrease in the recovery period. The use of exercise transcutaneous pO_2_ has recently gained interest in claudication, specifically in case of atypical claudication or claudication of questionable vascular origin [3]–[5]. We recently reported that, in patients with claudication and assumed peripheral artery disease referred for constant-load walking tests, the transcutaneous pO_2_ changes while walking could be automatically classified into 4 empirically predefined types [6]. Two of them mimic the expected physiological walking-induced increase in arterial pO_2_ (type A & B). One is based on a progressive decrease of transcutaneous pO_2_ throughout exercise with a progressive post-exercise recovery, as should be observed during exercise-induced hypoxemia. The last type shows an abrupt decrease at walking onset, a stabilization (or slow increase) throughout the walking period and an abrupt overshoot in the early recovery period. This last specific profile type was referred to as a “walking-induced transient hack” profile [6]. The transcutaneous pO_2_ changes are reliable in test-retest procedures in the same patient [6]. The proportion of each of these 4 transcutaneous pO2 types was almost identical in two distinct populations of patients referred for the diagnosis or follow-up of peripheral artery disease. The last two profile types (C & D) are assumed to be abnormal responses to walking.

Two major limitations characterized our previous work. First, the observed transcutaneous pO_2_ changes were compared to subjectively and empirically predefined models that were based on the expertise of the laboratory clinicians. Second, transcutaneous pO_2_ changes could have been due to local mechanisms (e.g., vasoconstriction, increased oxygen consumption, abnormal diffusion) that interfered with the estimation of arterial pO_2_ changes from transcutaneous pO2 changes. Therefore, our first aim in the present study was to apply an observer-independent clustering analysis to a new series of chest transcutaneous pO_2_ recordings in a new population of subjects, to assess whether this new analytic approach results in a classification comparable to our initial analysis. The second aim, an essential step for future studies of physiopathology, was to determine whether the transcutaneous pO_2_ hacks are associated with comparable underlying changes in systemic arterial pO_2_ and thus whether they reflect a walking-induced transient hypoxemia.

## Methods

### Participants

Both study 1 and study 2 were performed in patients referred to the Laboratory for Exercise Investigation and Sport Medicine of the University Hospital in Angers (France).

### Test Methods

#### STUDY 1

As a laboratory routine, all patients referred for claudication to the laboratory had a short physical visit in which history and treatments were recorded and body characteristics (age, gender, stature, weight) were measured. We retrospectively analyzed all 245 consecutive new patients that were referred over a 1 year period (January to December 2011). These patients were different from those included in our previous study [6]. This retrospective analysis of our laboratory routine results and the observational study did not require patient consent or registration, according to French law 2004-806 on biomedical research. Data is reported in accordance with the STAndards for the Reporting of Diagnostic accuracy Studies (STARD) guidelines [7].

In brief, the exercise tests were performed in a temperature-controlled room (21±2°C) after an acclimatization period of at least 20 minutes, on a pre-calibrated motor-driven treadmill (EF1200; Tecmachine, France) at a constant load (3.2 km/h; 10% slope with the transition phase from 0 to 3.2 km/h lasting 1 minute). Transcutaneous pO_2_ measurements were performed using a TCM400 (Radiometer DK) with the chest electrode on the right suprascapular region (except in case of history of thoracotomy). Once the electrode was in position, a pre-test heating period of at least 15 minutes was required to let resting values stabilize. Transcutaneous pO_2_ was recorded for 2 minutes in the standing position before the treadmill started. Patients who walked for 15 minutes (∼750 m) were considered non-limited and were excluded from the studies. After walking cessation, a 10-min recovery period was recorded. Our laboratory standard technique classifies patients into 4 groups by cross-correlating each patient curve to 4 empirically predefined types [6]. The cross-correlation coefficient matches the chest transcutaneous pO_2_ changes with the four most frequent types of curves that we have previously found: types 1, 2, 4 & 9. For practical reasons, in the present study we use the labels “A”, “B”, “C” and “D” instead of the original model types 1, 2, 4 and 9, respectively. Each patient was classified into the group for which the chest transcutaneous pO_2_ changes showed the best coefficient, provided that this coefficient was greater than 0.65. As in our previous study, a minimal decrease of 3 mmHg was required to classify the patient as type C or D to avoid misclassification due to transient artifacts [6].

Recordings obtained in patients analyzed with our laboratory standard technique were re-analyzed statistically using clustering, a technique commonly used in data analysis [8]–[13]. For the clustering approach, within each final class, a curve was obtained by averaging all waveforms belonging to each patient.

Also called “unsupervised classification”, clustering can be defined as the organization of patterns into coherent groups. Clustering automatically discovers and identifies groups (called clusters) in a data set, such that data in each cluster are similar and dissimilar data belong to different clusters. In the clustering analysis and after, we assumed that we had a data set *X* of *p*-dimensional space **R** *^p^*. Clustering techniques group data without *a priori* knowledge of the classes. Thus, to obtain the best realizations with techniques using distance criteria [14], we give some mathematical definitions. Clustering partitions the data set *X* into a given number of *_T_* subsets (clusters), with *C_i_*, 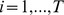. After the clustering processes, the *_T_* final clusters must respect the following conditions:
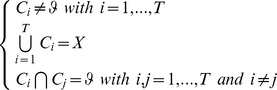
(1)


**Figure 1:**
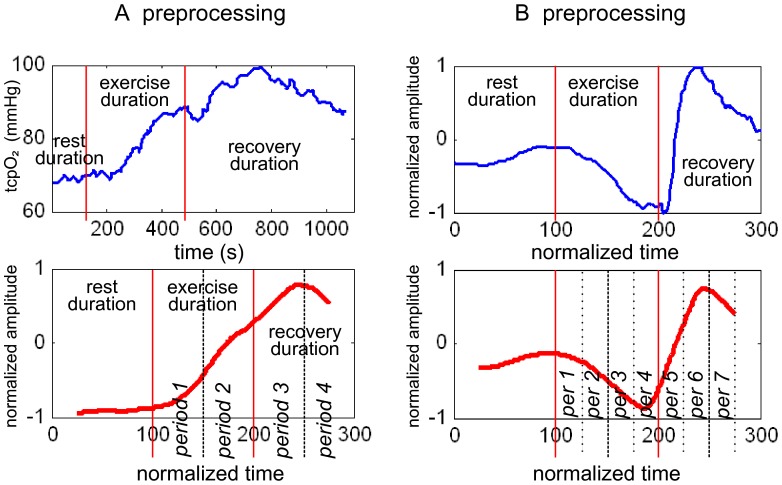
Preprocessing results. (a) The upper graph shows the original transcutaneous pO_2_ signal. The lower graph shows the preprocessed transcutaneous pO_2_ signal before clustering (second step) and the periods where the waveform is cut. (b) The upper graph shows the waveform of a cluster after the first step of the clustering. The lower graph shows the preprocessed waveform.

For the present study, each sample (Transcutaneous pO_2_ chest measurement) was characterized by a waveform (figure 1a, upper graph). Because walking duration was different among subjects, to improve the clustering analysis as a first step, we performed preprocessing (figure 1a, lower graph): each waveform was resampled by the linear interpolation technique to obtain 100 samples for each duration (rest, exercise and recovery). Finally, each waveform was denoised by a simple moving average with a window size of 25 data points (figure 1, lower graph).

As a second step, the merging of the waveforms into clusters was based on proximity (or similarity) measurement, and to improve this merging, we extracted several relevant features from the waveform that characterized them. The problem can be considered a time-series analysis, and the key step in this analysis involves transforming the original value into a stream of discretized symbols [15]. Thus, our features represented the slope of the waveform for four main periods (exercise and recovery durations); in figure 1a (lower graph), these periods are noted period*_i_*, 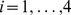. In the same figure, the symbolic representation of the waveform slopes is “**⇑, ⇑, ⇑,** ⇓“ (corresponding to the cluster “o” in table 1). As slopes can be positive or negative there are 2^4^ = 16 possible clusters (table 1). Irrelevant clusters (clusters that included no or only one waveform) were removed from the analysis. Within each cluster, a curve was obtained by averaging all waveforms belonging to each cluster.

**Table 1:** Characteristics of patients showing assumed normal (A or B) or abnormal (C or D) tcpO_2_ profile types or that could not be classified.

	TypeA or B	TypeC or D	Nonclassifiable	P
	*n = 140*	*n = 87*	*n = 18*	
Gender (males)	112 (80.0%)	75 (86.2%)	13 (72.2%)	NS
Age (years)	65.3+/−10.9	64.0+/−13.5	67.9+/−10.2	NS
Stature (cm)	168+/−8	171+/−7	166+/−9	*$
Body mass (kg)	77.3+/−16.5	83.0+/−14.1	73.9+/−18.1	*
Body mass index (kg/m^2^)	27.2+/−4.7	28.5+/−4.5	26.9+/−5.9	NS
Hemoglobin (g/dl)	14.1+/−1.7	14.1+/−1.7	13.6+/−2.1	NS
Resting saturation (%)	98+/−1	98+/−2	98+/−1	NS
*(Number of available data)*	*(n = 133)*	*(n = 84)*	*(n = 18)*	
Ankle-to-brachial index	0.68+/−0.23	0.77+/−0.22	0.71+/−0.30	*
Active smokers	44 (31.4%)	26 (29.9%)	3 (16.7%)	NS
MWT (sec)	237+/−187	273+/−221	287+/−198	NS
Reported pulmonary disease	30 (21.4%)	29 (33.4%)	0 (0%)	*

* is p<0.05 between type A or B and type C or D;

$ is p<0.05 between type A or B and non classifiable; NS is no significant difference.

The third step was to reduce the number of clusters obtained at step two to a limited number of classes. For this purpose, we transformed the average waveforms of relevant clusters by a symbolic representation according to the slope in different periods. However, in this step, we defined the slopes more exactly. On the one hand, we considered 7 periods of 25 data points each (per*_i_*, 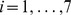): 4 in the exercise and 3 in the recovery period (figure 1b). For each period, the difference “Dp” between the value of the 25^th^ and the 1^st^ point was calculated. We encoded the changes as “2^−^” for Dp<−2/175; “1^−^” for −2/175< Dp<−1/175, “0” for −1/175<Dp<+1/175; “1^+^” for 1/175< Dp<2/175”; and “2^+^” for Dp>2/175. In figure 1b, the symbolic representation is “1^−^, 2^−^, 2^−^, 1^−^, 2^+^, 2^+^, 2^−^”.

**Figure 2:**
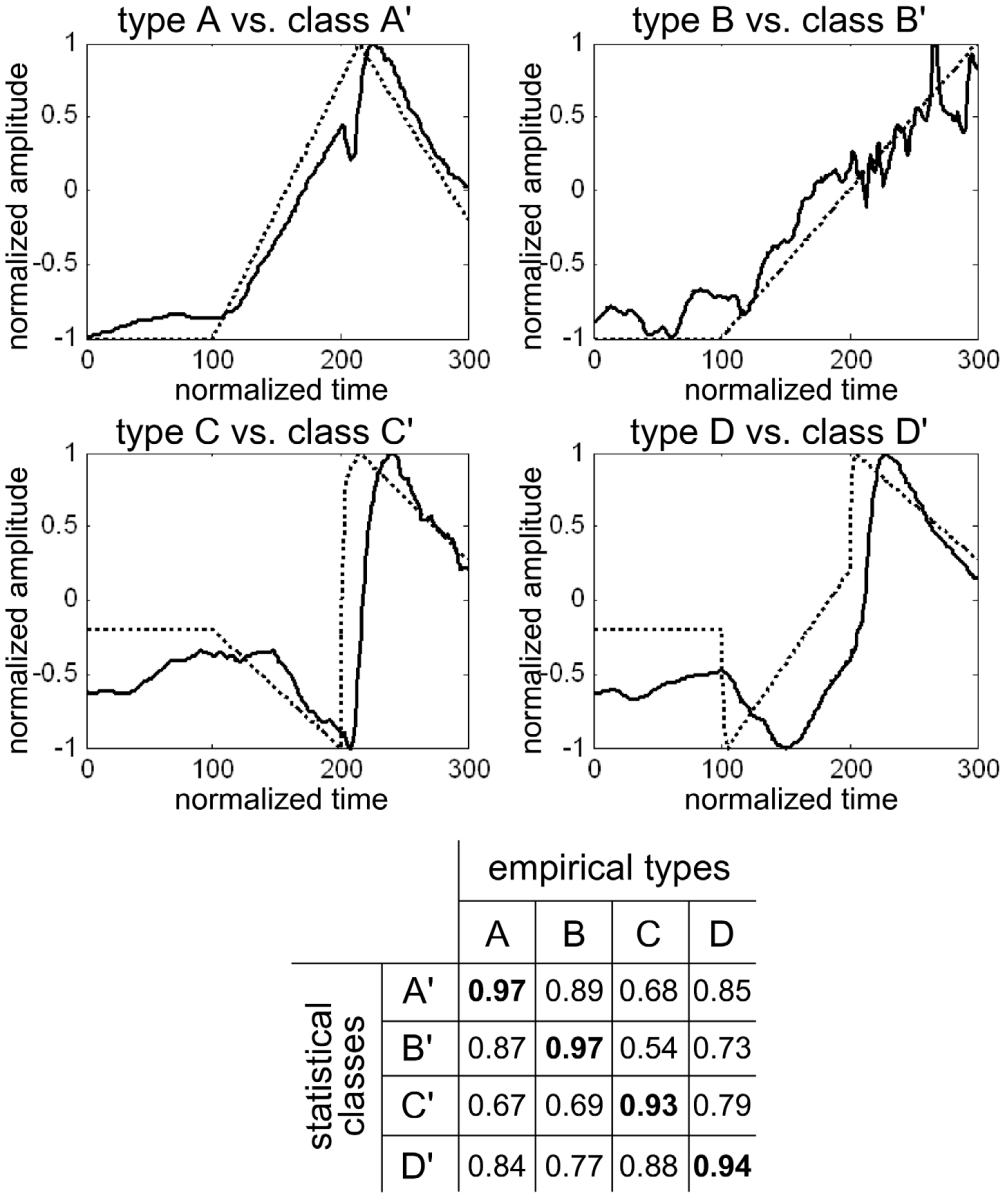
Comparison between the empirical models and the statistical models resulting from the clustering analysis. Empirical models are dotted lines, and statistical models are solid lines. In the figure, the exercise period is in gray. In the table, the highest coefficients are in bold characters.

Thereafter, merging of initial relevant clusters into classes was achieved by hierarchical agglomerative clustering (HAC). This approach determines successive levels of classes from an initial set of pre-established clusters until obtaining a single class. At each level, it organizes the classes by merging the two most similar clusters or classes. This method organizes the classes into a hierarchical structure according to a proximity matrix. The proximity matrix represents the similarity measures between all pairs of clusters. The similarity index constructing the classes depends on the distances between the symbolic representations of the previous level’s clusters and is based on a rank correlation index. The process of hierarchical clustering is represented in figure 2 by a tree structure called a dendrogram: it shows how the clusters have been grouped together level by level. The partition of the final classes is obtained by choosing the cutoff on the dendrogram. To optimize the cutoff, we computed a score based on the ratio of the between-class variance to the within-class variance for each step of the HAC process. This score can be interpreted like Fisher’s criterion [16], where the optimal partition maximizes and minimizes, respectively, the between-class and within-class variances. This resulted in 4 mathematically defined classes A′, B′, C′ and D′. Within each class, a curve was obtained by averaging all waveforms belonging to each patient of the class.

As for our laboratory routine, each patient was classified in the class for which the cross-correlation coefficient with the clustering model was the highest, provided that the coefficient was at least 0.650. Otherwise, the patient curve was considered non-classifiable. The clustering analysis was performed by one investigator (M Feuilloy) blinded to the results of the laboratory routine classification.

#### STUDY 2

We performed a prospective study among adult patients, referred for claudication, who showed hacks on their chest transcutaneous pO_2_ profile on the treadmill during the laboratory routine. Patients with no limitation on the treadmill (walking distance >500 m) or an ankle–brachial index <0.90; those who were under protection by law; those who were unable to understand the purpose of the study; or patients having a non-patent arterial palmary anastomosis, a history of gut disease, anticoagulant or antiplatelet treatment, a history of hand surgery, life-threatening disease, or known or documented cardiopulmonary disease were not eligible. Eligible patients were asked to participate and received a full explanation orally and in writing about the goals and risks of the study. To be included, each participant had to sign a consent form to participate after full explanation of the protocol. This study was approved by the local IRB, “CPP-Ouest II”, funded and promoted by the university hospital and registered before inclusion of the first patient in the ClinicalTrials.gov database (NCT01022606). Twenty-eight of the 38 eligible patients refused to participate. After inclusion, an indwelling catheter was inserted into the radial artery under local anesthesia by a trained anesthetist (C Dussaussoy), and then a second walking test was performed. Arterial blood was sampled at rest; at least at one, two and three minutes during exercise and just before the end of exercise; and at one and two minutes into the recovery period and at the end of the recovery period. Arterial samples were maintained on ice and analyzed within 30 minutes for arterial pO_2_ using a blood-gas analyzer calibrated with tonometered blood (ABL5™, Radiometer; Copenhagen, Denmark). Body temperature changes during exercise were measured with a telemetric system (Vitalsens Cortemp; USA). The temperature-sensitive pill was swallowed by the patient approximately thirty minutes before each test to allow stabilization of baseline values. Arterial pO_2_ was corrected for in vivo temperature changes using standard procedures [17]. Clinical control of radial permeability was performed systematically after the test.

### Statistical Methods

In study 1, the mean curve of each class was compared to the empirical graph type using the cross-correlation technique. Kappa statistics were used to compare patients’ classifications resulting from the statistical and empirical models. Generally, a kappa above 0.75 or 0.80 indicated adequate agreement between two classifications [18], [19]. Adjusted Wilcoxon statistics were used to quantify variation in study 2. The results are presented as the mean ± SD or median [25th–75th centiles] where appropriate in the text and as the mean ± SEM in figures. Statistical analyses were performed with SPSS version 13.0 software for windows. A two-tailed P<0.05 was used to indicate statistical significance.

## Results

In study 1, we analyzed all the patients who had been investigated in the laboratory from January to November 2011. The patients were 200 males and 45 females; age: 65±12 yrs; stature: 169±8 cm; weight: 79±16 kg; body mass index: 27.6±4.8 kg/m^2^; hemoglobin: 14.1±1.7 g/dl; resting pulse oximetry: 98±1%; lowest ankle-to-brachial index (n = 235 due to non-compressible arteries or missing data): 0.72±0.23; 73 active smokers. All had stable symptoms for a minimum of 3 months. Their maximal walking time on the treadmill was 189 [122–304] sec. Fifty-nine of the 245 patients (24.1%) reported or were treated for chronic respiratory diseases. Characteristics of patients with A or B profile type (assumed normal responses), C or D profile type (assumed abnormal responses) and with profiles that could not be classified are reported in table 1. As shown, few differences are found between the three groups. As expected, there were more patients reporting pulmonary disease in groups C or D than A or B, but no difference in the type of disease was found.

**Figure 3:**
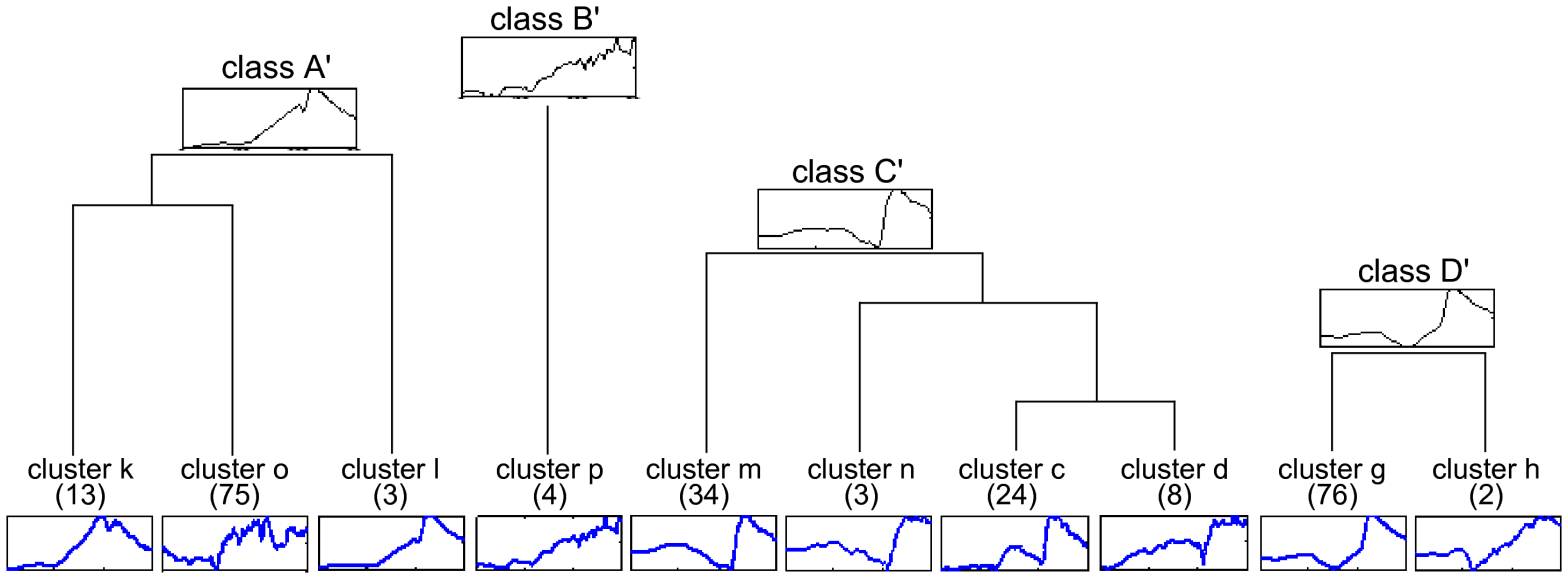
Dendrogram representation for hierarchical clustering of clusters.

The decomposition of the initial 245 profiles observed in the patients resulted, after the first two steps of the analysis, in 16 clusters (table 2). Several clusters (“a”, “b”, “e”, “f”, “i” and “j”) were not relevant (one or no patients). Therefore, the hierarchical agglomerative clustering was based on clusters “c”, “d”, “g”, “h”, “k, “l”, “m”, “n”, “o” and “p”. Finally the mean models of relevant clusters were issued from mean curves within four classes, called A′, B′, C′, and D′ (figure 3).

**Table 2:** Distribution of waveforms into the initial 16 clusters.

Cluster	A	b	c	d	e	F	g	h	i	j	k	l	m	n	o	p
Period 1	⇓	⇓	⇓	⇓	⇓	⇓	⇓	⇓	**⇑**	**⇑**	**⇑**	**⇑**	**⇑**	**⇑**	**⇑**	**⇑**
Period 2	⇓	⇓	⇓	⇓	**⇑**	**⇑**	**⇑**	**⇑**	⇓	⇓	⇓	⇓	**⇑**	**⇑**	**⇑**	**⇑**
Period 3	⇓	⇓	**⇑**	**⇑**	⇓	⇓	**⇑**	**⇑**	⇓	⇓	**⇑**	**⇑**	⇓	⇓	**⇑**	**⇑**
Period 4	⇓	**⇑**	⇓	**⇑**	⇓	**⇑**	⇓	**⇑**	⇓	**⇑**	⇓	**⇑**	⇓	**⇑**	⇓	**⇑**
Number	0	1	24	8	0	1	76	2	1	0	13	3	34	3	75	4

Figure 2 shows the similarity between the final waveforms of the mathematical classes and the empirical types. The cross-correlation coefficients between types and classes are presented in figure 2. When the initial 245 profiles were tested against the statistical models resulting from the clustering analysis, 18 profiles could not be classified because the cross-correlation with models A′, B′, C′ & D′ were <0.650 in all cases. The results for the remaining 227 profiles are presented in table 3. The comparison between the empirical types and cluster analysis-induced classes is shown in table 3. Cohen’s kappa of the data in table 2 was 0.865.

**Table 3:** Confusion matrix for classification by the statistical models versus the empirical models.

		Empirical types				
		A	B	C	D	Total
Statistical classes	A′	104	20	0	1	125
	B′	1	9	0	0	10
	C′	0	0	31	0	31
	D′	5	1	17	38	61
	Total	110	30	48	39	227

The beginning and end dates of recruitment for study 2 were November 2009 and April 2011, respectively. Ten patients agreed to participate. The patients were 8 males and 2 females; 60±14 yrs; 169±13 cm; 80±15 kg; one active smoker; hemoglobin: 15.6±1.8 g/dl; resting pulse oximetry: 98±2%. All included patients were symptomatic for a minimum of 1 year. Three patients reported dyspnea; the other seven reported lower limb claudication, among which only two also complained of a history of dyspnea. Their maximal walking time on the treadmill was 245 [192–545] sec. No patient had known cardiac or pulmonary dysfunction. Three patients had diabetes, and four were diagnosed with moderate peripheral artery disease. No major bleeding occurred at the catheter measurement site, but one patient had an occlusion of the radial artery that persisted for one week after the test but was relatively well tolerated (moderate sensation of a cold hand).

**Figure 4:**
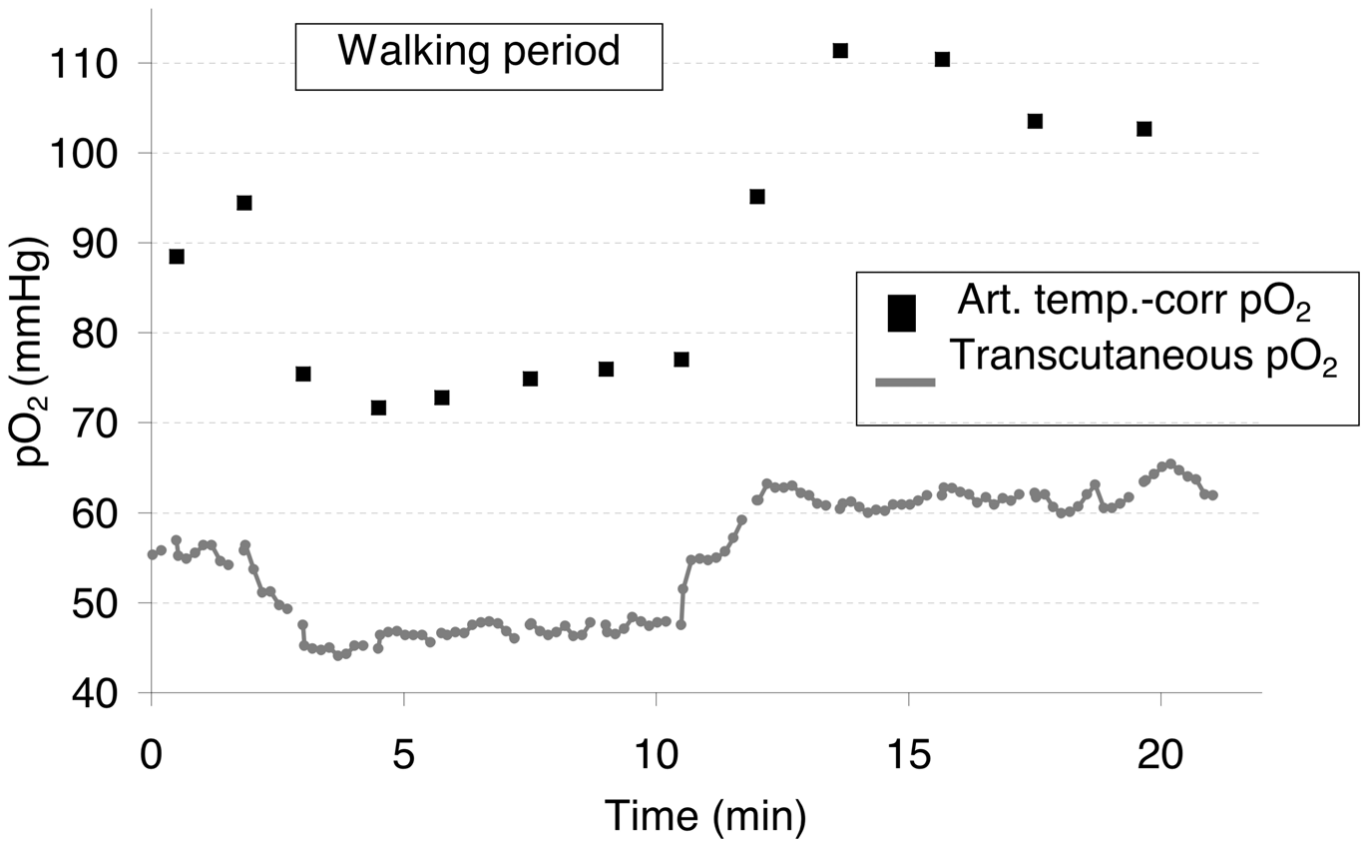
Simultaneous recording of chest transcutaneous and arterial oxygen pressure. Chest transcutaneous oxygen pressure (Transcutaneous pO_2_) and arterial pressure corrected for temperature changes (Art. temp.-corr. pO_2_). The dark square is the walking period.

Typical results from a patient showing a chest transcutaneous pO_2_ hack are presented in figure 4, showing the initial decrease and abrupt post-exercise overshoot.

**Figure 5:**
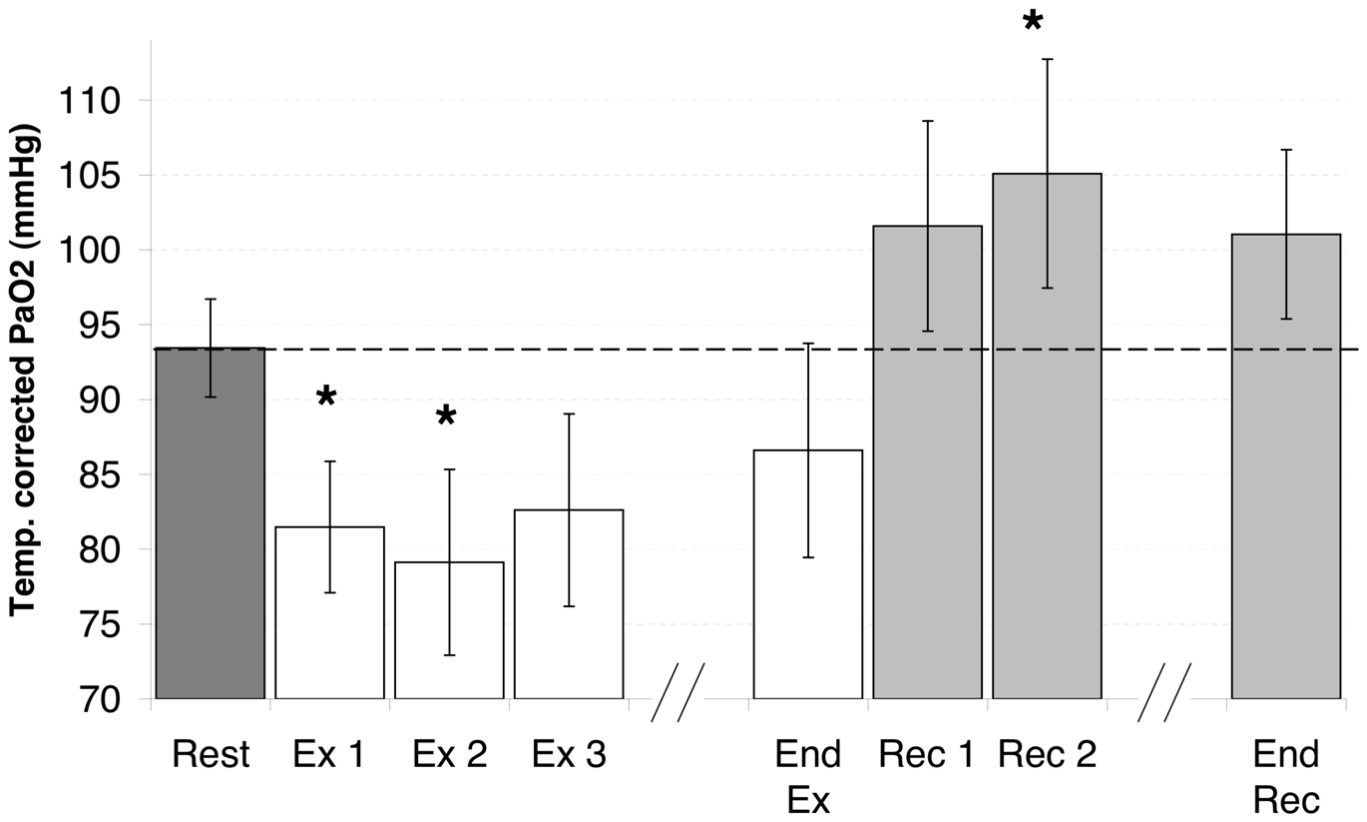
Mean ± SEM of temperature-corrected arterial oxygen pressure. Ex1, Ex2, Ex3 and End Ex are minutes 1, 2, 3 and the end of exercise. Rec1, Rec2 and End Rec are minutes 1, 2 and the end of recovery. *p<0.05 vs. rest.

On average (figure 5), arterial pO_2_ was in the normal range at rest and reached a minimum at two minutes of exercise. The decrease in pO_2_ was partly restored just before the exercise ended and reached a maximum in the first minutes of recovery. The analysis of variance showed a significant change (p<0.01) from baseline at 1 and 2 minutes of exercise and at two minutes into the recovery period.

## Discussion

The major results of the present study can be summarized as follows.

1. The observed models resulting from a mathematical observer-independent clustering analysis in a relatively large group of patients different from the one used in our previous study [6] produced 4 groups that closely fit the groups produced by the arbitrary models based on the expertise of the clinicians. This further validates the fact that profiles can be classified into only four groups.
2. In most eligible patients who underwent direct blood sampling, hack profiles on chest transcutaneous pO_2_ were associated with arterial walking-induced transient hypoxemia, occurring at exercise onset, and with a recovery overshoot. This confirms that in these patients the changes observed were due to a walking-induced transient hypoxemia.

As previously suggested [6], among the four types of chest transcutaneous pO_2_ changes, types A and B (and then classes A′ and B′) are assumed to reflect the normal arterial pO_2_ physiological changes. Types C & D (classes C′ & D′) are assumed to occur in patients with abnormal exercise response. The proportion of patients in groups C and D was much higher than in our previous study [6]. Assuming that the profiles of types C and D are abnormal, one-third of the patients whom we studied had an abnormal oximetric response to walking. The prevalence of pulmonary disease is high in patients with cardiovascular disorders [20], [21], but all respiratory diseases do not lead to hypoxemia. According to Von Kemp, 16% of patients considered for arterial surgery have chronic obstructive pulmonary disease, newly diagnosed in 3.5% of them, and 4.5% have unexpected disorders, which are all new diagnoses [21]. We assume that the high proportion of profiles of types C and D (or classes C′ and D′) result from the interest of our laboratory in unexplained walking impairment and atypical claudication. This proportion would most likely be lower in a general population or in non-exercise-limited patients. Still, **7%** of patients in the study 1 failed to be included into the groups derived from the clustering analysis. This could be interpreted as an unsatisfactory or insufficient classification procedure results from the predefined minimal cross-correlation coefficient used as a cut-off to avoid misclassification. Whether this cut-off point could be lowered for future studies remains to be studied.

Transcutaneous oximetry has been used for years in an attempt to non-invasively estimate underlying tissue pO_2_ in humans. Unfortunately, although the transcutaneous pO_2_ absolute value in neonates reasonably correlates with the arterial pO_2_ absolute value, this is not the case in adults. An unpredictable transcutaneous gradient exists between tissue and surface transcutaneous pO_2_. As a result, the estimation of the arterial pO_2_ absolute value by the transcutaneous pO_2_ absolute value is inadequate in adults. Second, the response of the transcutaneous pO_2_ probes in case of abrupt changes in the pO_2_ to be measured is slow, with 90% of the response times in the range of 30 to 40 sec [22]. Therefore, transcutaneous pO_2_ is inadequate to detect very fast (and/or short-lived) changes in the pO_2_ to be measured. Last, as a surface skin measurement, despite local heating of probes, changes in regional perfusion (regional ischemia, cutaneous vasoconstriction, etc.) may interfere with the relationship of transcutaneous pO_2_ with underlying tissue pO_2_, resulting in transcutaneous pO_2_ being much lower than the value to be recorded. Nevertheless, if the aim is to estimate relatively slow arterial pO_2_ changes, regardless of absolute values, changes in transcutaneous pO_2_ will mimic changes in arterial pO_2_ in normally perfused areas. Thereby, analyzing chest transcutaneous pO_2_ changes over time (regardless of the starting absolute value) may provide some estimation of underlying arterial pO_2_ changes over time, provided that the tissue changes are “reasonably” slow [1], [2]. Although previous studies show convincing results as to the validity of transcutaneous pO_2_ to estimate arterial pO_2_ changes regardless of absolute starting values [23], it could be suggested that the hack profiles result from an artifact that interferes with transcutaneous estimation of arterial pO_2_ changes in our specific technical situation. The reference technique to identify exercise-related hypoxemia is direct arterial puncture to measure arterial pO_2_. Direct sampling was not performed systematically in all our patients, either for technical and security reasons or because many patients refused the direct sampling. Nevertheless, when performed, the tests showed that the transcutaneous pO_2_ profile was confirmed in the arterial sampling in most cases. This clearly refutes the idea that the transcutaneous hacks could result from exercise-induced cutaneous vasoconstriction interfering with transcutaneous pO_2_ measurements. Overall, the test gradually intensifies over the first minute and is unlikely to induce a prolonged and severe vasoconstriction.

There are limitations to this study:

First, we provide no definitive proof in the present study that the presence of a hack (type D or class D′) is an abnormal response to walking and is the cause of walking limitation. Preliminary results from the laboratory seem to confirm that most of these patients have unsuspected respiratory diseases. Consistently, although profile types are reliable in test-retest recordings [6], future studies should evaluate the effect of treatments on chest transcutaneous pO_2_ profile (i.e., if the presence of a hack is abnormal, does it persist after treatment of an eventually associated pulmonary disease?).

Second, one could suggest that transcutaneous pO_2_ measurement is a complex and expensive procedure compared to pulse oximetry. If moderate exercise, such as walking, is done, in healthy Caucasian subjects (mean age 20–50 years), resting and walking pulse oximetry values are 98±0.9% and 97±1.3%, respectively [24]. Limitations of pulse oximetry to detect hypoxemia result from the sigmoid relationship between oxygen arterial saturation and pressure, specifically if starting arterial pO_2_ is high. Furthermore, if the minimal arterial pO_2_ value occurs at exercise onset, it is likely that pulse oximetry will only show a transient decrease that can hardly be differentiated from a recording artifact.

Third, we studied a specific population and a single specific exercise procedure. Chest transcutaneous pO_2_ changes should be evaluated in other groups of patients or during various exercise procedures.

Fourth, it is clear that due to the risk and invasive nature of study 2, many eligible patients refused to participate, and a recruitment bias cannot be excluded.

Lastly, we have no evidence for the underlying mechanisms of the pO_2_ changes observed. The pooling of blood with poor oxygen content in varicose veins [25], [26] due to prolonged standing before the start of exercise is a possible explanation. Exercise-induced intrapulmonary arterio-venous shunting could also have occurred. This was suggested as the underlying mechanism of exercise-induced hypoxemia in healthy humans [27], [28] but is a subject of debate [29], and it occurs primarily during heavy exercise. Differences in the kinetics of muscle blood flow changes to muscle oxygen consumption at the onset [30] and offset of exercise with aging or in diseased states could be one explanation, and future studies are required to test this. Finally, transiently improved cardiac output [31] after exercise, as reported in patients with chronic heart failure, could have contributed to a transient arterial pO_2_ overshoot by improving the ventilation/perfusion ratio at the chest level in the early recovery period.

Despite those limitations, our findings have potential major clinical interest. Despite the recommendations that during incremental (but possibly not constant-load, as used here) exercise, an arterial catheter should be used [32], in most laboratory routines, only two measurements of arterial pO_2_ are performed, and these involve small needles [33]: one at rest and one at end-exercise. In patients showing a walking-induced transient hypoxemia, arterial pO_2_ will most likely not appear to be decreased at end-exercise. It may even appear increased (and, as such, consistent with the expected normal response) if the sole “exercise sample” is in fact obtained in the early recovery period. This is what occurs when peak exercise measurement cannot be obtained due to movements or puncture difficulties. Therefore, the transient hypoxemia resulting from exercise will remain undetected. To accurately detect the initial arterial hypoxemia, arterial samples should be systematically obtained not only at rest and just before the end of exercise but also (i) in the first minute of exercise to detect the initial fall, (ii) throughout the walking period, (iii) a few seconds after exercise is stopped to detect the overshoot and (iv) at the end of the recovery period. The problem with these samplings is the need for multiple arterial punctures or the use of an indwelling arterial catheter, resulting in a significant number of local complications [34]. An alternative solution would be to select patients who should have multiple arterial punctures with exercise chest transcutaneous pO_2_, while only two samples are likely sufficient in most patients, specifically if chest transcutaneous pO_2_ changes are not consistent with a hack profile. Recording of near infrared spectroscopy (NIRS) at the forehead should be tested as another alternative solution to estimate systemic changes in oxygen concentration [35].

### Conclusion

Objective classification of chest transcutaneous pO_2_ changes based on clustering analysis closely matches the classification based on previously established, arbitrarily based curves, but 7% of patients failed to be included into the groups derived from the clustering analysis. Walking-induced transient hacks on chest transcutaneous pO_2_ recordings do reflect a walking-induced transient hypoxemia occurring at exercise onset. We think that in patients with unexplained walking impairment, chest transcutaneous pO_2_ recording during walking could help to detect unusual arterial pO_2_ changes during exercise and to select those patients for whom a sole peak-exercise (end-exercise) sample might be insufficient.
